# Selective estrogen receptor modulators inhibit growth and progression of premalignant lesions in a mouse model of ductal carcinoma *in situ*

**DOI:** 10.1186/bcr1317

**Published:** 2005-09-13

**Authors:** Ruria Namba, Lawrence JT Young, Jeannie E Maglione, Erik T McGoldrick, Stephenie Liu, Gregory T Wurz, Michael W DeGregorio, Alexander D Borowsky, Carol L MacLeod, Robert D Cardiff, Jeffrey P Gregg

**Affiliations:** 1 Department of Pathology and Laboratory Medicine, School of Medicine, University of California, Davis, Sacramento, CA, USA; 2 Department of Internal Medicine, Division of Hematology and Oncology, School of Medicine, University of California, Davis, Sacramento, CA, USA; 3 Center for Comparative Medicine, University of California, Davis, CA, USA; 4 Department of Medicine, UCSD Cancer Center, School of Medicine, University of California, San Diego, La Jolla, CA, USA

## Abstract

**Introduction:**

Ductal carcinoma *in situ *(DCIS) is a noninvasive premalignant lesion and is considered a precursor to invasive carcinoma. DCIS accounts for nearly 20% of newly diagnosed breast cancer, but the lack of experimentally amenable *in vivo *DCIS models hinders the development of treatment strategies. Here, we demonstrate the utility of a mouse transplantation model of DCIS for chemoprevention studies using selective estrogen receptor modulators (SERMs). This model consists of a set of serially transplanted lines of genetically engineered mouse mammary intraepithelial neoplasia (MIN) outgrowth (MIN-O) tissue that have stable characteristics. We studied the ovarian-hormone-responsiveness of one of the lines with a particular focus on the effects of two related SERMs, tamoxifen and ospemifene.

**Methods:**

The estrogen receptor (ER) status and ovarian-hormone-dependence of the mouse MIN outgrowth tissue were determined by immunohistochemistry and ovarian ablation. The effects of tamoxifen and ospemifene on the growth and tumorigenesis of MIN outgrowth were assessed at 3 and 10 weeks after transplantation. The effects on ER status, cell proliferation, and apoptosis were studied with immunohistochemistry.

**Results:**

The MIN-O was ER-positive and ovarian ablation resulted in reduced MIN-O growth and tumor development. Likewise, tamoxifen and ospemifene treatments decreased the MIN growth and tumor incidence in comparison with the control (*P *< 0.01). Both SERMs significantly decreased cell proliferation. Between the two SERM treatment groups, there were no statistically significant differences in MIN-O size, tumor latency, or proliferation rate. In contrast, the ospemifene treatment significantly increased ER levels while tamoxifen significantly decreased them.

**Conclusion:**

Tamoxifen and ospemifene inhibit the growth of premalignant mammary lesions and the progression to invasive carcinoma in a transplantable mouse model of DCIS. The inhibitory effects of these two SERMs are similar except for their effects on ER modulation. These differences in ER modulation may suggest different mechanisms of action between the two related SERMs and may portend different long-term outcomes. These data demonstrate the value of this model system for preclinical testing of antiestrogen or other therapies designed to prevent or delay the malignant transformation of premalignant mammary lesions in chemoprevention.

## Introduction

Improvements in mammography screening now permit the detection of early breast lesions such as ductal carcinoma *in situ *(DCIS). These lesions represent the most rapidly growing subgroup of breast cancers and comprise nearly 20% of all newly diagnosed cases of breast cancer. Although DCIS is considered a noninvasive lesion, if it is left untreated, invasive carcinoma will develop in 40% to 50% of DCIS cases [1]. Thus, effective treatment of DCIS could substantially reduce the incidence of invasive breast cancer. However, developing strategies for DCIS treatment has been difficult, partly because of a lack of an experimentally amenable *in vivo *model.

Over the past several years, our group has developed and characterized a mouse model for DCIS that shares biological, morphological, and molecular characteristics with human DCIS [2,3]. In addition, this model meets the criteria for mouse premalignant mammary intraepithelial lesions set by the NIH Annapolis Pathology Panel of the Workshop on Mouse Models of Human Breast Cancer [4]. The definition of mammary intraepithelial neoplasia (MIN) is based on morphological criteria and the biological behavior of the lesions assessed by ectopic and orthotopic transplantation in syngeneic recipient mice. A MIN lesion is incapable of ectopic growth but capable of orthotopic growth (in a gland-cleared mammary fat pad), and this growth has a consistently high rate of transformation to 'malignancy', defined as the ability to grow in ectopic and orthotopic locations.

The model described here was derived by transplanting focal mammary lesions from mouse mammary tumor virus (MMTV) polyomavirus middle T (PyV-mT) transgenic mice to syngeneic wild-type host mammary fat pads. The Tg(*PyV-mT*) model is an attractive human breast cancer model not only because of its molecular similarities to the human breast cancer, but also because of the similar morphology and histology to human breast cancer [5]. *PyV-mT *is a membrane-bound viral oncogene. The signaling pathways activated by *PyV-mT *include those of Ras, Shc, and phosphotidylinositol 3-kinase, which are frequently activated in human breast cancer (reviewed in [6]) and are also activated by *ErbB2 *(*Her2/neu*), a receptor tyrosine kinase that is overexpressed in 30% of breast cancer and is associated with poor outcome [7]. The molecular profile of Tg(*PyV-mT*) mouse mammary tumors is more similar to that of *Neu/ErbB2 *and *myc *transgenic mouse mammary tumors than to other transgenic mouse tumors [8]. The histology of Tg(*PyV-mT*) mammary tumors resembles Tg(*Neu/ErbB2*) mammary tumors and human breast cancer much more closely than other transgenic mammary tumors [9]. Stages of Tg(*PyV-mT*) mammary tumor development also recapitulate human breast cancer progression histologically as well as in the expression of biomarkers associated with poor prognosis [5].

Stable mammary intraepithelial neoplasia outgrowth (MIN-O) lines have been established by microscopically identifying and dissecting premalignant dysplastic foci from Tg(*PyV-mT*) mammary fat pads and serially transplanting them into the gland-cleared mammary fat pads of wild-type FVB/N host mice [2,10]. The transplanted MIN tissue grows to fill the host mammary fat pad, and after a certain latency period, tumor foci arise within the MIN-Os. The lines have been maintained over several years by serial transplantation of MIN-Os to new host fat pads. Therefore, the MIN-O lines provide the biology of the tumor progression found in the original Tg(*PyV-mT*) mammary fat pad in an experimentally reproducible setting.

Estrogen exposure is an important breast cancer risk factor. Seventy percent of breast cancers express the estrogen receptor (ER) [11]. The absence of ER in breast cancer is associated with poor prognosis. In the Tg(*PyV-mT*) model, relatively high numbers of ER-positive cells are found in the early MIN stage, but invasive tumorigenesis is associated with loss of ER [5]. In a Tg(*PyV-mT*) tumor explant model with a low to moderate expression of ER, tumor grew slower in ovariectomized animals than in intact animals, whereas estrogen supplementation stimulated rapid tumor growth, suggesting that this model is sensitive to estrogen level [12].

In ER-positive human breast cancer, a selective estrogen receptor modulator (SERM), tamoxifen, is typically used in adjuvant therapy. The US Food and Drug Administration (FDA) has approved tamoxifen, and studies have shown that in ER-positive cancer, treatment for 5 years reduces recurrence by 47% and the risk of death by 26% [13]. Unfortunately, tamoxifen therapy is associated with undesirable side effects, including endometrial cancer, thromboembolic events, and liver cancer (as seen in animal models [14]) as well as hot flashes, insomnia, vaginal discharge, and vaginal dryness [15]. Therefore, the search for other SERMs has continued. Recently, third-generation aromatase inhibitors, which block the conversion of androgens to estrogens, have been shown to be potentially more efficacious than tamoxifen [15], but recently reported side effects, including bone loss, joint pain, and cardiac events, have caused concern [16].

Therefore, a model that recapitulates human DCIS, with progression to invasive carcinoma with demonstrated ER-positivity and estrogen-dependency would allow for preclinical studies to assess the efficacy of this new generation of SERMs and aromatase inhibitors. Here we have used one transplantable MIN-O line, 8w-B, because it has a relatively short tumor latency, has uniform histopathology, has consistent molecular architecture over multiple transplant generations, and is ER-positive [3]. To further explore the ovarian-hormone-dependency of the MIN-O line, growth of the MIN-O transplant and tumor incidence were determined after ovariectomy. To demonstrate the utility of this model in antiestrogen therapy, we treated the animals with tamoxifen, the gold standard and FDA-approved adjuvant therapeutic for ER-positive cancer, and a less extensively studied SERM, ospemifene. Ospemifene (FC-1271a; Z-2-[4-(4-chloro-1,2-diphenyl-but-1-enyl)phenoxy]ethanol), is structurally similar to tamoxifen but has a more benign side-effect profile, including proestrogenic in the bone and neutral in the endometrium [17]. It is currently in phase III clinical trials for the urogenital sequelae, but there are limited data demonstrating its effectiveness in breast cancer. In our study, both tamoxifen and ospemifene treatments resulted in a similar level of suppression of the MIN-O growth and tumor incidence. In addition, Ki-67, a marker for proliferation and a potential efficacy biomarker, showed decreased expression with treatment. Interestingly, we found that tamoxifen treatment decreased the ER status of the MIN-Os while ospemifene treatment did not. This suggests that these two related SERMs act differently and that there may be differential long-term effects. Overall, these studies show that this MIN model can be used for preclinical trials and is a suitable preclinical model for antiestrogen therapy.

## Materials and methods

### Mice

Standard techniques for mammary gland clearing and transplantation, and the establishment and characterization of the MIN-O line, have been described previously [2]. Three-week-old FVB/J female mice were purchased from Charles River Laboratories (Wilmington, MA, USA). The surgery and treatments were carried out in the animal housing facility on the University of California Davis campus following the approved procedures.

### Ovariectomy study

8w-B premalignant MIN-O tissue (1 mm^3^) [2] was transplanted in the gland-cleared no.4 fat pads of 3-week-old virgin FVB/J female mice. In addition, at the time of transplantation, the experimental group (8 animals) was ovariectomized. The control group (4 animals) received the transplants and sham surgery without ovariectomy. The tumor latency was measured by weekly palpation. The extent of MIN-O growth was assessed at 5 weeks after transplantation by exposing the transplanted mammary fat pad and visually inspecting the transplanted MIN-Os under a dissecting microscope. The animals were palpated weekly until a tumor was detected. The experiment was concluded at day 99.

### Chemoprevention study

SERM treatments were prepared by suspending ospemifene (Hormos Medical Corporation, Turku, Finland) or tamoxifen citrate (Orion Corporation, Orion-Pharma, Espoo, Finland) in dimethyl sulfoxide (DMSO) and then diluting with peanut oil to the final concentration of 1% DMSO. Animals received 50 mg/kg of tamoxifen or ospemifene in 100 μl peanut oil daily by oral gavage. These concentrations have been shown to be effective in mice (MW DeGregorio, unpublished results). The control group received 100 μl of 1% DMSO in peanut oil.

Three-week-old gland-cleared FVB/J female mice were treated with tamoxifen, ospemifene, or vehicle control starting 1 week before the MIN-O transplantation, ensuring therapeutic levels of the SERM at the time of transplantation. In the first experiment, animals (*n *= 4 per group) were sacrificed 3 weeks after transplantation. In the second experiment, animals (*n *= 9 for tamoxifen, *n *= 12 for ospemifene, and *n *= 13 for control) were sacrificed 10 weeks after transplantation. For both experiments, at the time of sacrifice, the MIN-O growth was recorded as gross percentage of area filled in the fat pad (% fat pad filled), and the tumor foci were measured in two dimensions under a stereomicroscope. Each fat pad was fixed in 10% formalin for histological analysis. Tumor incidence was assessed from histological sections of each fat pad. In addition, serum was obtained from each animal for HPLC analysis. At autopsy, each mouse was assessed for any other abnormalities.

### Histology

Methods for whole-mount preparation of fixed mammary fat pads and immunohistochemistry on the paraffin-embedded fat pads have been described previously [2]. The following primary antibodies were used with the VECTASTAIN ABC Elite Kit (Vector Laboratories, Burlingame, CA, USA): rabbit anti-ER (1:600, LabVision, Fremont, CA, USA), anti-Ki-67, anti-cleaved caspase-3 (1:250, Promega, Madison, WI, USA), and anti-PyV-mT (B4Rat7; 1:50; Dr Gernot Walter, UC San Diego).

ER positivity was scored according to the method of Harvey and colleagues [18]. The frequency of ER-positive cells was scored on a scale of 0 to 5, where 0 = no ER staining, 1 = up to 1% of cells with ER positivity, 2 = 1% to 10%, 3 = 11% to 33% (one-third), 4 = 34% to 66%, and 5 = more than two-thirds of cells with ER positivity. The intensity of staining was scored on a scale of 0 to 3, where 0 = no staining, 1 = weak staining, 2 = intermediate staining, and 3 = strong staining. The final ER score was calculated by adding the frequency score and the intensity score. ER staining in proliferating edge and differentiated center zone was scored separately for each MIN-Os (*n *= 17 for control, *n *= 16 for ospemifene, and *n *= 11 for tamoxifen).

For quantification of Ki-67 and cleaved caspase-3 staining, the positive nuclei were counted and the total epithelial area was measured in Image-Pro PLUS (MediaCybernetics, Silver Spring, MD, USA) from at least four different 40×-objective images from each treatment group. The number of positive nuclei was normalized to the epithelial area of each image.

### HPLC analysis

Ospemifene and tamoxifen serum samples were quantified using a previously published assay [19,20]. Toremifene citrate (5 μg/ml in methanol (Orion Corporation, Orion Pharma, Espoo, Finland) was used as the internal standard for the ospemifene samples, and nafoxidine hydrochloride (50 μg/ml in methanol; Sigma, St Louis, MO, USA) served as the internal standard for the tamoxifen and control group samples.

### Statistical analysis

An unpaired *t*-test was used for the MIN-O size analysis in the ovariectomy and SERM treatment experiments and immunohistochemistry analysis. For the analysis of tumor latency in the ovariectomy study, a log-rank test was used. For the tumor incidence analysis, Fisher's exact test was used. All statistical analysis was performed using GraphPad Prism version 4.00 for Windows (GraphPad Software, San Diego, CA, USA).

## Results

### MIN-O lesions are ovarian hormone-sensitive

**Figure 1:**
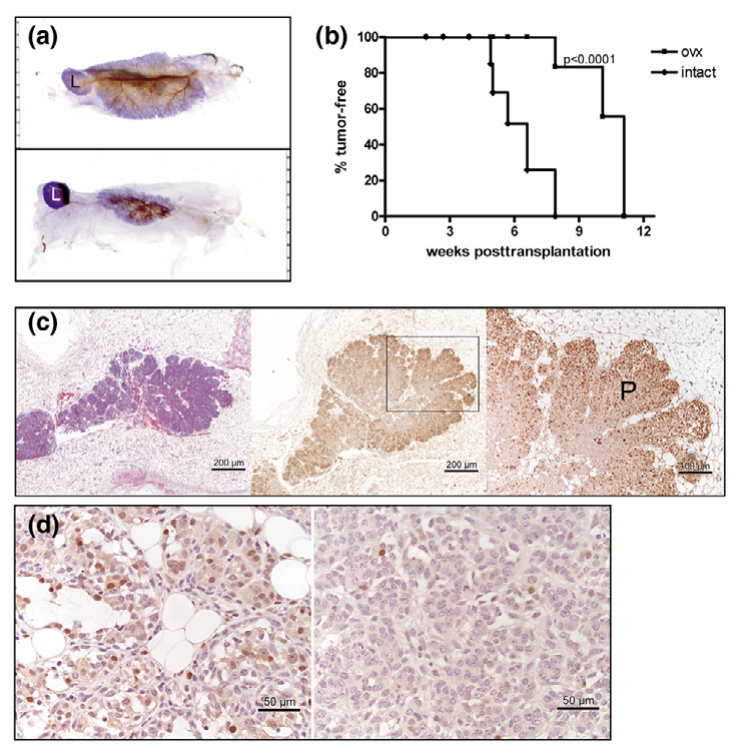
Effect of ovarian hormone on the development of mammary intraepithelial neoplasia outgrowths (MIN-Os) and tumors. **(a) **Whole-mount images, from intact (top panel) and ovariectomized (bottom panel) murine host mammary fat pads into which 8w-B premalignant MIN-O tissue had been transplanted. MIN-O (dark blue) is significantly larger in the intact host fat pad at 5 weeks after transplantation than in the ovariectomized host fat pad. Lymph node (L) is seen at the left side of the MIN-O. **(b) **Effect of ovarian ablation on the 8w-B MIN-O line tumor development. Time to palpable tumor was significantly longer in ovariectomized mice (*n *= 8) than in the intact ones (*n *= 4). **(c) **Immunohistochemical staining of the proliferation marker Ki-67 on MIN-O (center and right). The growing edge of the MIN-O is highly proliferative, as seen by intense Ki-67 staining (brown, 10× field, center). A 20× field of the boxed area is shown on the right. The proliferative area (P) is indicated. Corresponding H & E staining is shown on the left. **(d) **Immunohistochemical staining of estrogen receptor (ER)-α on MIN-O (left) and tumor (right) from 8w-B-line animals at 10 weeks after transplantation. Areas with strong nuclear ER staining as well as cytoplasmic staining were often found in the MIN-O tissue (left). In general, ER staining in tumor tissue was less intense and less frequent (right). ovx, ovariectomized.

The ovarian-hormone-dependence of the MIN-O was assessed by ovariectomizing the host FVB/J females. The sizes of the MIN-Os were measured as percentage of fat pad filled 5 weeks after transplantation. The MIN-Os in the ovariectomized animals were one-third the size of those in the control animals (*P *< 0.0001; Table 1; Fig. 1a) and tumor incidence was decreased (100% tumor free) as compared to the nonovariectomized controls (25% tumor free) at 5 weeks after transplantation. The ovariectomy prolonged the tumor latency of the 8w-B MIN-O line significantly (*P *< 0.0001) from TE_50 _(the time for 50% of the transplanted mammary fat pads to produce palpable tumors) of 6.6 weeks to 10.1 weeks (Table 1; Fig. 1b).

Since ER status is an important classifier of breast cancer, ER expression of the MIN-Os and tumors arising from the MIN-Os was analyzed by immunohistochemistry. Immunochemically detected ER positivity was most strongly present in the area closest to the proliferating edge of the MIN-Os (proliferating zone; Fig. 1c), where large clusters of ER-positive cells were found (Fig. 1d left), while in the center of the MIN-Os, where cells are more differentiated, ER-positive cells were present but more scattered. In the tumors, there were fewer ER-positive cells and these were distributed in a random fashion that has been seen in Tg(*PyV-mT*) tumors (Fig. 1d, right) [5]. In ovariectomized animals, ER expression remained without obvious change in intensity and distribution in the MIN-O tissues (data not shown).

### The effect of ospemifene and tamoxifen on MIN-O tumorigenesis

**Figure 2:**
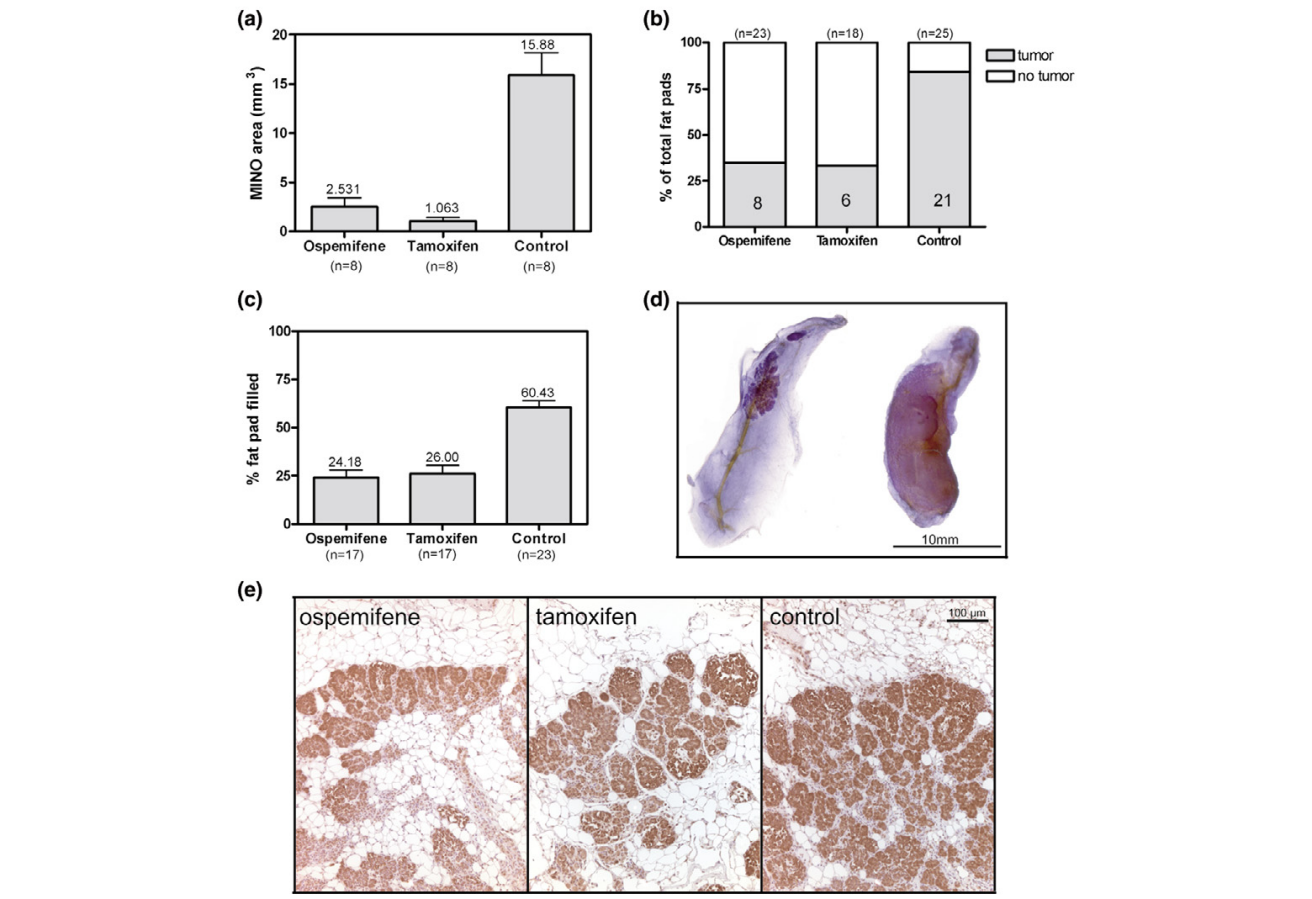
Effect of the selective estrogen receptor modulator (SERM) treatments. **(a) **Relative amount of mammary intraepithelial neoplasia outgrowth (MIN-O) tissue at 3 weeks after transplantation. The amount of MIN-O tissue in the fat pads of mice treated with ospemifene or tamoxifen was significantly less than that of untreated mice (*P *< 0.0001). **(b) **Tumor incidence of the MIN-O-transplanted fat pads at 10 weeks after transplantation. The actual number of tumor-bearing fat pads is shown within each bar. Tumor incidence in SERM-treated fat pads was significantly lower than in the untreated fat pads (*P *= 0.0009 for ospemifene and *P *= 0.0012 for tamoxifen). **(c) **MIN-O size at 10 weeks after transplantation. The ospemifene-treated and tamoxifen-treated MIN-Os were significantly smaller than the untreated MIN-Os 10 weeks after treatment (*P *< 0.0001). **(d) **Whole mounts of ospemifene-treated (left) and untreated (right) mammary fat pads at 10 weeks after transplantation. The untreated fat pad has a larger MIN-O area (dark blue). **(e) **Polyomavirus middle T (PyV-mT) expression in the MIN-Os was detected by immunohistochemistry with anti-PyV-mT antibody. Strong cytoplasmic expression of PyV-mT remained after the SERM treatment.

The effect of SERMs on the MIN-O development was evaluated by treating MIN-O-transplanted animals with either tamoxifen or ospemifene. In order to determine the effect on the MIN-O growth, rather than the effect on tumor incidence, SERM-treated animals were humanely sacrificed at 3 weeks after transplantation, before tumors typically develop. While all MIN-O transplants, regardless of the treatment type, had clearly increased in size from the initial transplant, the SERM-treated animals had significantly smaller MIN-Os than the untreated animals (*P *< 0.0001; Fig. 2a). On the other hand, the MIN-O sizes were not statistically different between the two SERM groups. At the time of termination at 3 weeks after transplant, no tumor foci were seen in any of the groups.

The effect of the long-term SERM treatment on tumorigenesis was evaluated at 10 weeks after transplantation (Fig. 2b–d). Typically, by 10 weeks the transplanted MIN-O will fill the majority of the fat pad, and tumor foci can be found within most of the MIN-Os. In this study, tumors were identified in 21 of the 25 control mammary fat pads, 8 of the 23 ospemifene-treated mammary fat pads, and 6 of the 18 tamoxifen-treated mammary fat pads at 10 weeks after transplantation (Fig. 2b). The tumor incidences in both SERM groups were significantly lower than in the control group. There was no significant difference in the tumor incidences between the two SERM-treated groups. Both SERM groups had a smaller mean tumor size (34 mm^3 ^ospemifene, 46 mm^3 ^tamoxifen) than the control group (167 mm^3^), a difference that approached statistical significance (*P *= 0.12 and 0.14, respectively), while there was no significant difference in mean tumor size between the SERM groups. Moreover, the MIN-O size was significantly smaller in both SERM groups than in the control group (*P *< 0.0001, Fig. 2c,d). The SERM treatment did not affect the intensity of the cytoplasmic PyV-mT staining in the MIN-O epithelium (Fig. 2e).

Both ospemifene and its major metabolite, 4-hydroxy-ospemifene, were found at biologically active concentrations in the serum from the ospemifene-treated animals, while tamoxifen and its major metabolite, *N*-desmethyl-tamoxifen, were found at biologically active concentrations in the tamoxifen-treated animals (data not shown). Ospemifene, tamoxifen, and their two metabolites were not detected in the control animals.

### Immunohistochemical analysis of SERM-treated MIN-Os and tumors

**Figure 3:**
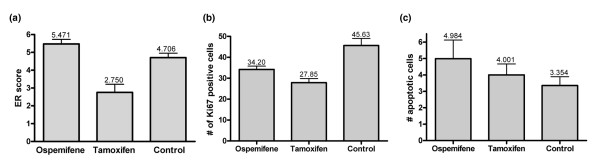
Immunohistochemistry analysis of mammary intraepithelial neoplasia outgrowths (MIN-Os) treated with selective estrogen receptor modulator (SERM). **(a) **Estrogen receptor (ER)-positive status was assessed by immunohistochemisty. ER positivity was slightly increased in ospemifene-treated MIN-Os (*P *< 0.05) and significantly decreased by tamoxifen treatment (*P *= 0.0005). **(b) **The cell proliferation rate in SERM-treated MIN-Os was assessed by counting nuclei positive for the proliferation marker Ki-67 in a 40× field. Cell proliferation was in general decreased by the SERM treatments (*P *< 0.05 for ospemifene and *P *< 0.01 for tamoxifen). **(c) **SERM treatments slightly increased the averaged apoptotic cell number in the MIN-Os but the differences were not statistically significant (*P *= 0.198 for ospemifene and *P *= 0.4768 for tamoxifen).

ER expression in the treated and untreated tissues was examined by immunohistochemistry. We found that the tamoxifen treatment resulted in decreased ER score (less intense and less frequent) in the proliferating edges of the MIN-Os (Fig. 3a). Diminished ER expression by tamoxifen treatment has previously been reported in a Wnt-1 model [21]. Ospemifene treatment, on the other hand, did not decrease the ER score; rather, it increased the number of ER-positive cells in the MIN-Os.

The architecture and morphology of the MIN-Os and tumors did not differ among the groups, despite the differences in the size of the lesions. SERM treatments, in general, resulted in decreased proliferation and increased apoptosis. SERM-treated MIN-Os retained the zone of increased proliferation at the MIN-O/stroma interphase, but there were significantly fewer Ki-67-positive cells in this zone in MIN-Os from the tamoxifen treatment (*P *< 0.01) and ospemifene treatment (*P *< 0.05) than in the control MIN-Os (Fig. 3b). The number of apoptotic cells, visualized with antibody against cleaved caspase-3, was increased in both ospemifene and tamoxifen-treated MIN-Os but the differences were not statistically significant (*P *= 0.198 and 0.4768 respectively, Fig. 3c).

## Discussion

The study reported here is to our knowledge the first chemoprevention study to compare the efficacy of two SERMs, using a transgenic mouse transplant model that fulfills the operational definition of premalignancy. We demonstrate that the transplanted premalignant lesions (MIN-Os) are ER-positive and their growth is ovarian-hormone-sensitive. Two related SERMs, ospemifene and tamoxifen, had an inhibitory effect on this mouse model for DCIS on the growth of premalignant mammary lesions and on the tumor incidence. The primary effect of these two agents was on proliferation, while apoptosis was less affected. In comparing the two SERMs, no significant differences were found in the growth of the premalignant lesion, tumor incidence, and markers for proliferation or apoptosis. In contrast, there was a significant difference in the ER status between the two SERM-treated groups. The ER score in the MIN-O lesions was reduced after treatment with tamoxifen but increased by treatment with ospemifene. Overall, these studies provide a foundation for studying and comparing the effects of hormonal manipulation in a chemoprevention setting in an immunocompetent transplantable mouse model for DCIS.

The MIN-O line, 8w-B, originally isolated from dysplastic lesions in the Tg(*PyV-mT*) mammary glands, was found to be ER-positive and ovarian-hormone-sensitive in this study. Recent studies with Tg(*PyV-mT*) mice have demonstrated that the distribution and number of ER-positive cells change with progression to malignancy [5]. In our studies, we observed a specific distribution of ER-positive cells in the MIN-Os, showing a higher ER score near the leading edge of the growing outgrowth. Tumors, on the contrary, typically had cells with weak ER positivity randomly scattered throughout the tumor. To functionally assess this observation that the MIN-O is estrogen-positive, the host animals were ovariectomized to study the effect on MIN-O growth and tumor incidence. Ovariectomy significantly reduced the growth of the MIN-O and it significantly increased the tumor latency. This data, coupled to the receptor levels, suggests that MIN-O growth and progression to invasive tumor is ovarian-hormone-dependent, albeit not exclusively. This is reminiscent of human breast cancer, in which oophorectomy decreases recurrence and the incidence of contralateral invasive breast cancer [22,23].

Both SERMs exerted an inhibitory effect on the premalignant tissue growth. As each initial transplant contains 1 mm^3 ^of tissue, the results at 3 weeks of treatment show that the transplants are growing, albeit at a much slower rate. In addition, this decreased growth effect is not due to decreased *PyV-mT *expression in the treatment groups, as the *PyV-mT *expression was not different between the SERM groups and the control group, based on immunohistochemistry.

The SERMs also inhibited progression to invasive carcinoma. Typically, a high proportion of the MIN-Os will have tumors by 10 weeks after transplantation. The SERM-treated animals showed diminished MIN-O growth with significantly fewer tumors than controls at this time point. The major effect of the SERMs was on proliferation as seen by Ki-67 staining, rather than on apoptosis. In human breast cancer, reduced Ki-67 staining is seen after treatment with SERMs and aromatase inhibitors and has been used as efficacy marker in antiestrogen therapy [24]. No statistically significant differences in MIN-O growth, tumor incidence, and rates of proliferation and apoptosis were observed between the two SERM treatments. One exception was the effect on ER expression. The ospemifene treatment increased the ER score, whereas tamoxifen decreased it. In ER-positive mammary tumors from Wnt-1 transgenic mice, tamoxifen treatment resulted in significant reduction of ER expression [21]. In a portion of human tumors, decreased ER levels in tumors after tamoxifen treatment has been shown to predict tamoxifen resistance [25]. Therefore, these differences in ER modulation between the two related SERMs may suggest different mechanisms of action and may portend different long-term outcomes. This may be reflected in the slight differences in cell proliferation and apoptosis rate between the two treatment groups.

The study detailed here is the first to compare these two related SERMs in a mouse mammary premalignant transplant model and shows that ospemifene has equal effects to tamoxifen in mammary lesions. This is important, because ospemifene is currently in phase III clinical trials for urogenital sequelae but limited data demonstrate its effectiveness in breast cancer. As phase I and II studies have shown it was well tolerated in healthy postmenopausal women, it may offer alternative hormonal options to women with breast cancer or at high risk for breast cancer. In particular, ospemifene is not known to cause menopausal symptoms, such as hot flashes, insomnia, [26,27], melancholy, nervousness, dizziness, while it has some proestrogenic effects on the bone [17] and vaginal tissue [26] and, unlike other SERMs, does not cause vaginal dryness. Recently, aromatase inhibitors have been shown to provide better chemoprevention to the breast than tamoxifen, but similar to tamoxifen, they have significant side effects including bone loss, muscle and joint sequelae, and cardiac events [15].

These studies provide evidence that both tamoxifen and ospemifene have effects on decreasing growth and progression in our model of DCIS. Previously, the chemopreventive effects of ospemifene have been studied in a dimethylbenz [a]anthracene (DMBA)-induced rat and mouse tumor models, where it reduced the incidence of mammary tumors [28]. Although the DMBA-induced models have been utilized by many investigators in chemoprevention studies, they have significant drawbacks in that they can only test the ability of an agent to affect the progression to invasive carcinoma, rather than examining the effects on the early preneoplastic disease. In addition, the tumors that do arise from DMBA treatment are commonly heterogeneous and involve many organs. Moreover, a large portion of breast carcinomas derived in DMBA-treated animals are adenocanthomas, which do not represent or model typical human invasive carcinoma [29]. The chemopreventive effects of tamoxifen have been studied in various mouse models, including transgenic mice with activated *neu *expression. In Tg(*MMTV-neuN*) mice, which exhibit estrogen-sensitive tumor development [30,31], tamoxifen treatment reduced the mammary tumor incidence and size when the treatment was initiated before subclinical tumors had developed [32,33]. More recently, tamoxifen was shown to delay tumorigenesis in an ER-positive Tg(*P53*^-/-^) mammary premalignant transplant model [34].

The MIN-O model illustrated in these studies offers many advantages over other mouse mammary carcinoma models for chemoprevention studies. In typical transgenic mouse mammary models, tumorigenesis occurs in a multifocal manner, that is, multiple tumor foci develop in a mammary fat pad arising independently and at different starting times. Thus, in a given mammary fat pad, multiple lesions at different stages of tumorigenesis can be seen. Since no two fat pads are the same with respect to the development of the lesions, interpreting the results of chemopreventative interventions can be very complicated. Moreover it may require a significant amount of animals to distinguish the effect of an intervention. In contrast, in the MIN-O model, the proliferation of the 'premalignant' growth begins upon transplantation, and therefore the time to malignant transformation is easily measurable. Chemopreventative interventions can be applied before transplantation, at transplantation, or at a defined time after the time of transplantation. In particular, line 8w-B, a MIN-O line used in this study, has a defined tumor latency period [2]. This relatively short latency affords the opportunity to perform chemoprevention experiments rapidly. Secondly, the premalignant MIN-O and the invasive tumor mimic the histopathology of, respectively, human DCIS and invasive tumor [2,10]. Third, since each experimental subject receives tissue from the same MIN-O, the comparison of the experimental and control groups is less prone to error due to differences in the biological potential of the tissue. Fourth, the outgrowths continue to maintain the same biological characteristics, such as tumor latency, histopathological characteristics, and molecular profiles, over multiple serial transplant generations [2,3]. This phenotypic stability affords the opportunity to compare experiments over time, regardless of the transplant generation.

## Conclusion

The MIN-O line has the necessary biological and functional characteristics to be utilized as a mouse model for preclinical chemopreventive studies for human DCIS. This model, in an immunocompetent animal, is ER-positive and progresses from premalignant disease to invasive carcinoma. In particular, we show that tamoxifen significantly reduces the growth rate and tumor incidence of the 8w-B line. More importantly, we also show that this model can be used to analyze a therapeutic agent for which we have little data with respect to its chemopreventive effects in the breast. A promising result emerging from this study is that ospemifene exhibits efficacy in breast chemoprevention comparable to that of tamoxifen. Therefore, this model provides a platform to investigate and compare the effectiveness of antiestrogen agents, both as single agents and potentially in combination with other synergistic agents.

## Abbreviations

DCIS = ductal carcinoma *in situ*; DMBA = dimethylbenz [a]anthracene; DMSO = dimethyl sulfoxide; ER = estrogen receptor; FDA = Food and Drug Administration; H & E = hematoxylin and eosin; HPLC = high-performance liquid chromatography; MIN = mammary intraepithelial neoplasia; MIN-O = mammary intraepithelial neoplasia outgrowth; MMTV = mouse mammary tumor virus; PyV-mT = polyomavirus middle T; SERM = selective estrogen receptor modulator.

## Competing interests

MD has patents on ospemifene and tamoxifen on subject matter not related to this manuscript and has received research funding within the past 5 years for projects unrelated to this manuscript. The authors declare that they have no other competing interests.

## Authors' contributions

RN carried out the SERM treatment studies, assisted with the ovarian ablation study, analyzed the data, and drafted the manuscript. LJTY carried out the transplantation and ovarian ablation surgeries and assisted with all the animal studies. JEM carried out the ovarian ablation study. LJTY and JEM also developed the MIN-O model. ETM carried out the transplantation surgeries and assisted with the SERM treatment studies. SL assisted with the animal studies and the drafting of the manuscript. GTW and MWD participated in the design of the SERM study and carried out the serum analysis. ADB and RDC assisted in the histology analysis and made contributions to the interpretation of data. CLM and RDC conceived of the MIN-O model and applications of the model and contributed to the drafting of the manuscript. JPG conceived and coordinated the study and helped to draft the manuscript. All authors read and approved the final manuscript.

## Figures and Tables

**Table 1:** Effect of ovarian ablation in mice on MIN-O growth in fat pads

Mice	MIN-O size at 5 weeks after transplantation			Tumor latency	
	%FPF (no.)	Relative to control	*P*	TE_50 _(weeks)	*P*

Control	67.50 ± 12.58 (4)	1	0.0001	6.60	0.0001
Ovariectomized	22.86 ± 7.56 (7)	0.34		10.10	

%FPF, percentage of fat pad filled; MIN-O, mammary intraepithelial neoplasia outgrowth; TE_50_, time for 50% of the transplanted mammary fat pads to produce palpable tumors.

## References

[B1] Cuzick J (2003). Treatment of DCIS – results from clinical trials. Surg Oncol.

[B2] Maglione JE, McGoldrick ET, Young LJT, Namba R, Gregg JP, Liu L, Moghanaki D, Ellies LG, Borowsky AD, Cardiff RD (2004). Polyomavirus middle T-induced mammary intraepithelial neoplasia outgrowths: single origin, divergent evolution, and multiple outcomes. Mol Cancer Ther.

[B3] Namba R, Maglione JE, Young LJ, Borowsky AD, Cardiff RD, MacLeod CL, Gregg JP (2004). Molecular characterization of the transition to malignancy in a genetically engineered mouse-based model of ductal carcinoma in situ. Mol Cancer Res.

[B4] Cardiff RD, Anver MR, Gusterson BA, Hennighausen L, Jensen RA, Merino MJ, Rehm S, Russo J, Tavassoli FA, Wakefield LM (2000). The mammary pathology of genetically engineered mice: the consensus report and recommendations from the Annapolis meeting. Oncogene.

[B5] Lin EY, Jones JG, Li P, Zhu L, Whitney KD, Muller WJ, Pollard JW (2003). Progression to malignancy in the polyoma middle T oncoprotein mouse breast cancer model provides a reliable model for human diseases. Am J Pathol.

[B6] Dilworth SM (2002). Polyoma virus middle T antigen and its role in identifying cancer-related molecules. Nat Rev Cancer.

[B7] Slamon DJ, Clark GM, Wong SG, Levin WJ, Ullrich A, McGuire WL (1987). Human breast cancer: correlation of relapse and survival with amplification of the HER-2/neu oncogene. Science.

[B8] Desai KV, Xiao N, Wang W, Gangi L, Greene J, Powell JI, Dickson R, Furth P, Hunter K, Kucherlapati R (2002). Initiating oncogenic event determines gene-expression patterns of human breast cancer models. Proc Natl Acad Sci USA.

[B9] Rosner A, Miyoshi K, Landesman-Bollag E, Xu X, Seldin DC, Moser AR, MacLeod CL, Shyamala G, Gillgrass AE, Cardiff RD (2002). Pathway pathology: histological differences between ErbB/Ras and Wnt pathway transgenic mammary tumors. Am J Pathol.

[B10] Maglione JE, Moghanaki D, Young LJ, Manner CK, Ellies LG, Joseph SO, Nicholson B, Cardiff RD, MacLeod CL (2001). Transgenic polyoma middle-T mice model premalignant mammary disease. Cancer Res.

[B11] Althuis MD, Fergenbaum JH, Garcia-Closas M, Brinton LA, Madigan MP, Sherman ME (2004). Etiology of hormone receptor-defined breast cancer: a systematic review of the literature. Cancer Epidemiol Biomarkers Prev.

[B12] Dabrosin C, Palmer K, Muller WJ, Gauldie J (2003). Estradiol promotes growth and angiogenesis in polyoma middle T transgenic mouse mammary tumor explants. Breast Cancer Res Treat.

[B13] (1998). Tamoxifen for early breast cancer: an overview of the randomised trials. Early Breast Cancer Trialists' Collaborative Group. Lancet.

[B14] Carthew P, Lee PN, Edwards RE, Heydon RT, Nolan BM, Martin EA (2001). Cumulative exposure to tamoxifen: DNA adducts and liver cancer in the rat. Arch Toxicol.

[B15] Howell A, Cuzick J, Baum M, Buzdar A, Dowsett M, Forbes JF, Hoctin-Boes G, Houghton J, Locker GY, Tobias JS (2005). Results of the ATAC (Arimidex, Tamoxifen, Alone or in Combination) trial after completion of 5 years' adjuvant treatment for breast cancer. Lancet.

[B16] Kalidas M, Brown P (2005). Aromatase inhibitors for the treatment and prevention of breast cancer. Clin Breast Cancer.

[B17] Morello KC, Wurz GT, DeGregorio MW (2002). SERMs: current status and future trends. Crit Rev Oncol Hematol.

[B18] Harvey JM, Clark GM, Osborne CK, Allred DC (1999). Estrogen receptor status by immunohistochemistry is superior to the ligand-binding assay for predicting response to adjuvant endocrine therapy in breast cancer. J Clin Oncol.

[B19] Taras TL, Wurz GT, Hellmann-Blumberg U, DeGregorio MW (1999). Quantitative analysis of Z-2-[4-(4-chloro-1,2-diphenyl-but-1-enyl)phenoxy]ethanol in human plasma using high-performance liquid chromatography. J Chromatogr B Biomed Sci Appl.

[B20] Taras TL, Wurz GT, DeGregorio MW (2001). In vitro and in vivo biologic effects of Ospemifene (FC-1271a) in breast cancer. J Steroid Biochem Mol Biol.

[B21] Zhang X, Podsypanina K, Huang S, Mohsin SK, Chamness GC, Hatsell S, Cowin P, Schiff R, Li Y (2005). Estrogen receptor positivity in mammary tumors of Wnt-1 transgenic mice is influenced by collaborating oncogenic mutations. Oncogene.

[B22] Crump M, Sawka CA, DeBoer G, Buchanan RB, Ingle JN, Forbes J, Meakin JW, Shelley W, Pritchard KI (1997). An individual patient-based meta-analysis of tamoxifen versus ovarian ablation as first line endocrine therapy for premenopausal women with metastatic breast cancer. Breast Cancer Res Treat.

[B23] Buchanan RB, Blamey RW, Durrant KR, Howell A, Paterson AG, Preece PE, Smith DC, Williams CJ, Wilson RG (1986). A randomized comparison of tamoxifen with surgical oophorectomy in premenopausal patients with advanced breast cancer. J Clin Oncol.

[B24] Dowsett M, Smith IE, Ebbs SR, Dixon JM, Skene A, Griffith C, Boeddinghaus I, Salter J, Detre S, Hills M (2005). Short-term changes in Ki-67 during neoadjuvant treatment of primary breast cancer with anastrozole or tamoxifen alone or combined correlate with recurrence-free survival. Clin Cancer Res.

[B25] Gutierrez MC, Detre S, Johnston S, Mohsin SK, Shou J, Allred DC, Schiff R, Osborne CK, Dowsett M (2005). Molecular changes in tamoxifen-resistant breast cancer: relationship between estrogen receptor, HER-2, and p38 mitogen-activated protein kinase. J Clin Oncol.

[B26] Rutanen EM, Heikkinen J, Halonen K, Komi J, Lammintausta R, Ylikorkala O (2003). Effects of ospemifene, a novel SERM, on hormones, genital tract, climacteric symptoms, and quality of life in postmenopausal women: a double-blind, randomized trial. Menopause.

[B27] Voipio SK, Komi J, Kangas L, Halonen K, DeGregorio MW, Erkkola RU (2002). Effects of ospemifene (FC-1271a) on uterine endometrium, vaginal maturation index, and hormonal status in healthy postmenopausal women. Maturitas.

[B28] Qu Q, Zheng H, Dahllund J, Laine A, Cockcroft N, Peng Z, Koskinen M, Hemminki K, Kangas L, Vaananen K (2000). Selective estrogenic effects of a novel triphenylethylene compound, FC1271a, on bone, cholesterol level, and reproductive tissues in intact and ovariectomized rats. Endocrinology.

[B29] Medina D, Warner MR (1976). Mammary tumorigenesis in chemical carcinogen-treated mice. IV. Induction of mammary ductal hyperplasias. J Natl Cancer Inst.

[B30] Yang X, Edgerton SM, Kosanke SD, Mason TL, Alvarez KM, Liu N, Chatterton RT, Liu B, Wang Q, Kim A (2003). Hormonal and dietary modulation of mammary carcinogenesis in mouse mammary tumor virus-c-erbB-2 transgenic mice. Cancer Res.

[B31] Hewitt SC, Bocchinfuso WP, Zhai J, Harrell C, Koonce L, Clark J, Myers P, Korach KS (2002). Lack of ductal development in the absence of functional estrogen receptor alpha delays mammary tumor formation induced by transgenic expression of ErbB2/neu. Cancer Res.

[B32] Menard S, Aiello P, Tagliabue E, Rumio C, Lollini PL, Colnaghi MI, Balsari A (2000). Tamoxifen chemoprevention of a hormone-independent tumor in the proto-neu transgenic mice model. Cancer Res.

[B33] Nanni P, Nicoletti G, De Giovanni C, Landuzzi L, Di Carlo E, Iezzi M, Ricci C, Astolfi A, Croci S, Marangoni F (2003). Prevention of HER-2/neu transgenic mammary carcinoma by tamoxifen plus interleukin 12. Int J Cancer.

[B34] Medina D, Kittrell FS, Hill J, Shepard A, Thordarson G, Brown P (2005). Tamoxifen inhibition of estrogen receptor-alpha-negative mouse mammary tumorigenesis. Cancer Res.

